# Ground Maneuvering Target Detection and Motion Parameter Estimation Method Based on RFRT-SLVD in Airborne Radar Sensor System †

**DOI:** 10.3390/s26020559

**Published:** 2026-01-14

**Authors:** Lanjin Lin, Yang Zhao, Yang Yang, Dong Cao, Haibo Wang, Linyan Liu, Xing Chen

**Affiliations:** Aerodynamics Research and Development Center Computation Aerodynamics Institute of China, Mianyang 621000, China

**Keywords:** maneuvering target detection, motion parameter estimations, range migration, Doppler ambiguity, Doppler frequency migration, radar sensor

## Abstract

**Highlights:**

**What are the main findings?**

- This paper presents a high-order phase compensation and parameter estimation algorithm designed for maneuvering targets, which effectively balances detection performance and computational efficiency.
- The proposed method achieves precise compensation of high-order range migrations without the need for exhaustive parameter search, thereby circumventing the blind speed sidelobe effect. Futhermore, the incorporation of a time-delay variable significantly improves the anti-noise capability of the approach.

**What is the implication of the main finding?**

- This algorithm introduces a novel motion compensation technique to address the challenges of range migration, Doppler frequency migration, and Doppler ambiguity in detecting weak maneuvering targets. It achieves precise range migration compensation with enhanced accuracy and robustness, forming a vital preprocessing step for high-quality SAR imaging.
- The algorithm supports simultaneous multi-target processing and holds promising application potential in fields such as airborne radar detection, maritime moving target indication, cooperative observation by UAV formations, and other fields.

**Abstract:**

This study focuses on the key challenges in detecting and estimating motion parameters of ground maneuvering targets for airborne radar sensors. The complex unknown motion states of the ground maneuvering target, including velocity, acceleration, and jerk, result in range migrations (RMs) and Doppler frequency migrations (DFMs). These effects severely degrade the long-time coherent accumulation performance of the airborne radar, thereby limiting the reliable detection and precise parameter estimation of maneuvering targets. To address this issue, a new detection and motion parameter estimation method based on the range frequency reversal transform (RFRT) and searching Lv’s distribution (SLVD), i.e., RFRT-SLVD, is proposed. Specifically, the third-order RM (TRM) and quadratic DFM (QDFM) are considered. The proposed method operates as follows: First, RMs are eliminated simultaneously via the RFRT operation, which multiplies the echo by its reversed data in the range frequency and slow-time domains, leveraging the symmetric equal-interval sampling property of the range frequency. Subsequently, a phase compensation function (PCF) related to the jerk is constructed to compensate the QDFM. Finally, the LVD is performed to remove residual DFMs and achieve effective signal energy accumulation. Additionally, the case of a fast-moving target with Doppler ambiguity is analyzed, and a method for estimating three motion parameters is provided. A key advantage of the proposed technique is its ability to directly compensate the RMs without requiring prior knowledge of the maneuvering target, while also avoiding the blind speed sidelobe (BSSL) effect. In comparison with existing algorithms, RFRT-SLVD achieves a balanced trade-off between parameter estimation performance and computational efficiency. Numerical analyses and experiments are conducted to validate the method, assessing its detection capability for ground maneuvering targets, Doppler ambiguity resolution in parameter estimation, computational complexity, and method applicability in multi-target scenarios.

## 1. Introduction

This study focuses on the key application of long-time coherent integration for airborne radar sensors. As an important research area in airborne radar sensor systems, ground maneuvering target detection holds significant theoretical and practical value in fields, such as target tracking, high-resolution imaging, and motion parameter estimation, with growing practical demands [1,2]. With ongoing advances in detection technology, modern targets are often characterized by long detection ranges and a low radar cross-section (RCS), resulting in weak radar echoes and significantly increasing the detection difficulty of the radar sensors [3]. In this context, extending the radar observation time has become an effective approach to enhance accumulated signal energy, which can improve the signal-to-noise ratio (SNR) and thereby boost the detection performance of radar sensors [4,5]. In practical applications, the motion characteristics of maneuvering targets exhibit significant complexity. In addition to velocity and acceleration, jerk is also an important component. For example, in high-mobility tactical maneuvers such as emergency evasion by tanks or high-speed turns by combat vehicles, the resulting jerk effect is particularly pronounced and cannot be ignored. These motion parameters induce range migrations (RMs) and Doppler frequency migrations (DFMs) during long-time coherent integration. These effects manifest specifically as linear RM (LRM), range curvature (RC), third-order RM (TRM), and high-order DFMs, all of which severely degrade and constrain the moving target detection (MTD) [6] capability of radar sensor systems against moving targets [7,8].

Over the past few decades, significant research efforts have been devoted to addressing the aforementioned challenges. As for LRM caused by the target’s velocity, several typical methods, such as the keystone transform (KT) [9,10], Radon–Fourier transform (RFT) [11,12], axis rotation MTD (AR-MTD) [13], scaled inverse Fourier transform (SCIFT) [14], and modified location rotation transform (MLRT) [15] have been developed. However, these methods may suffer from accumulation degradation since they fail to adequately compensate for the RM and DFM caused by the target’s acceleration. To jointly mitigate both the effects of RM and DFM, a number of advanced algorithms have been introduced, e.g., the Radon-fractional Fourier transform (RFRFT) [16], Radon linear canonical transform (RLCT) [17], improved axis rotation–time reversal transform (IAR-TRT) [18], extended KT (EKT) [19], and deramp–keystone processing (DKT) [20]. Despite their effectiveness, these methods are generally limited to compensating for low-order motion parameters. Actually, maneuvering targets with complex dynamics often exhibit significant jerk motion, which brings about TRM and quadratic DFM (QDFM) during the observation time. Such higher-order effects can no longer be neglected, rendering the aforementioned techniques insufficient in such scenarios.

To address the effects of range migration (RM) and Doppler frequency migration (DFM) in maneuvering targets with complex motions, researchers have proposed solutions from various perspectives. Examples include parameter search-based algorithms such as the improved axis rotation–discrete chirp-Fourier transform (IAR-DCFT) [21], KT–Generalized Dechirp Process (KT-GDP) [22], KT–cubic phase function (KT-CPF) [23], generalized RFT (GRFT) [24], generalized KT and generalized dechirp process (GKTGDP) [25], and KT–Second-dimensional Matched Filtering Processing (KT-SMFP) [26].

IAR-DCFT first employs IAR to compensate RMs, followed by the application of the DCFT [27] to achieve coherent integration. Based on the detected peak position, the target’s velocity, acceleration, and jerk can be estimated. However, the IAR-DCFT necessitates a high-dimensional search over motion parameters, leading to a prohibitive computational burden. To alleviate the computational complexity, Li et al. present KT-GDP and KT-CPF. Both methods first apply the keystone transform to correct LRM. Subsequently, KT-GDP eliminates DFMs by constructing a two-dimensional (2-D) matched filtering function. This approach reduces computational load by decreasing the number of search parameters. In contrast, KT-CPF utilizes CPF [28,29] to remove DFMs, thereby reducing search dimensionality from two to one and further enhancing computational efficiency. Nonetheless, the above-mentioned algorithms ignore the high-order RMs, which may result in integration loss. To address high-order RMs, Xu et al. introduced the GRFT, which can compensate RMs and DFMs via a four-dimensional (4-D) search in the target’s motion parameter space. However, limited by the system’s pulse repetition frequency (PRF), the GRFT algorithm suffers from velocity ambiguity due to discrete pulse sampling. This indicates that signal energy can form not only a peak at the target’s true velocity but also a noticeable peak at ambiguous velocities (i.e., blind speeds), as the Doppler phase at blind speeds can still be effectively compensated. Although constrained by range resolution, the echo energy corresponding to blind speeds is weakened compared to that of the target, yet it still generates a significant blind speed sidelobe (BSSL), thereby resulting in a high false alarm rate. To effectively suppress BSSL, GKTGDP and KT-SMFP have been developed. These techniques eliminate RMs using the KT and apply matched filtering processing to remove residual coupling effects. Similarly to the GRFT, these two algorithms also involve high-dimensional parameter searches. Furthermore, the KT operations employed in these methods can introduce Doppler ambiguity and target trajectory splitting phenomena [26]. Through the above analyses, it can be seen that the computational complexity of multi-dimensional searching algorithms is high, which limits their practicality in engineering applications. In order to handle this conundrum, several methods to reduce the order of the polynomial phase are recommended. Such algorithms can effectively decrease the search dimensionality through a series of nonlinear operations. For instance, approaches based on the adjacent cross-correlation function (ACCF) [30] correct RMs and DFMs through two nonlinear operations; however, they suffer from limited estimation accuracy due to error propagation. To mitigate such propagation errors, the algorithm named time-reversing transform–second KT–Lv’s distribution (TRT-SKT-LVD) [31] is adopted. TRT-SKT-LVD first eliminates LRM, TRM, and QDFM by a multiplication operation. Then, the RC is corrected according to the SKT [32]. Finally, coherent integration is accomplished with LVD [33]. Attentively, although these three algorithms reduce the computational amount, the nonlinear operations may bring about performance degradation and are susceptible to cross-term interference.

It is evident that there exists an inherent trade-off between detection capability and computational efficiency, which has posed a challenge in the design of long-time coherent integration algorithms. Inspired by previous researches and with consideration of both detection capability and computational complexity, this paper proposes a novel algorithm for maneuvering target detection and high-order motion parameter estimation, termed range frequency reversal transform (RFRT) and searching LVD (SLVD), i.e., RFRT-SLVD. The proposed method first employs the RFRT, which performs a multiplication operation based on uniform sampling in the range frequency domain, to directly decouple the relationship between range frequency and slow time, thereby effectively eliminating the effects of LRM, RC, and TRM. After that, a phase compensation function (PCF) is constructed to dispel QDFM resulting from jerk motion. Finally, the LVD transform is applied to obtain the outcome of coherent accumulation and the estimations of motion parameters, including velocity, acceleration, and jerk. Notably, a comprehensive analysis is conducted regarding the impact of Doppler ambiguity for a fast-moving target on the algorithm’s velocity estimation. It should be noticed that this work is an extended and enhanced version of the conference paper [5]. Key additions include an analysis of multi-target scenarios, as well as a comprehensive evaluation of detection and parameter estimation performance via comparative simulations. Crucially, to resolve the velocity ambiguity problem in fast-moving targets, this paper proposes a method for estimating the true, unambiguous velocity.

Distinguished from the above-mentioned methods referenced in [6,7,8,9,10,11,12,13,14,15,16,17,18,19,20,21,22,23], the proposed algorithm accounts for the influence of jerk motion on both TRM and QDFM and is capable of effectively compensating for first-, second-, and third-order RM and DFM. In contrast to the GRFT, the proposed algorithm employs the RFRT to compensate RMs without a parametric search, thereby significantly reducing computational complexity. Furthermore, it achieves reduced-order processing of the higher-order Doppler phase via complex multiplication. This eliminates the need for discrete velocity searching and Doppler phase matching, as required in the GRFT. Consequently, the proposed mechanism circumvents the velocity ambiguity caused by discrete velocity sampling and effectively suppresses the BSSL. When compared with the ACCF iteratively method, the introduction of a time-delay variable via the LVD operation enhances coherent integration gain and improves robustness against noise. Moreover, unlike TRT-SKT-LVD, the proposed method effectively suppresses interference of cross terms. It should be noted that the algorithm proposed in this paper can effectively correct RMs through a single processing step via the RFRT, whereas the TRT-SKT-LVD algorithm requires two steps to handle LRM, RC, and TRM. More importantly, the TRT operation, while eliminating LRM and TRM, also removes the information of the target’s velocity and jerk. As a result, it cannot achieve parameter estimation. Overall, the recommended RFRT–SLVD offers a favorable compromise between computational efficiency and detection ability, taking both aspects into systematic consideration. Theoretical analyses and simulation results are presented to demonstrate the effectiveness of the proposed algorithm.

The remainder of this paper is organized as follows. In Section 2, the mathematical model of the radar received signal is established. In Section 3, we introduce the proposed maneuvering target detection algorithm and the parameter estimation of the method. In Section 4, we analyze the performance of the method with multiple targets, a quantitative analysis of the SNR, and computational complexity. In Section 5, some numerical experiments are provided. In Section 6, some conclusions and possible future research tracks are given.

## 2. Signal Model

**Figure 1:**
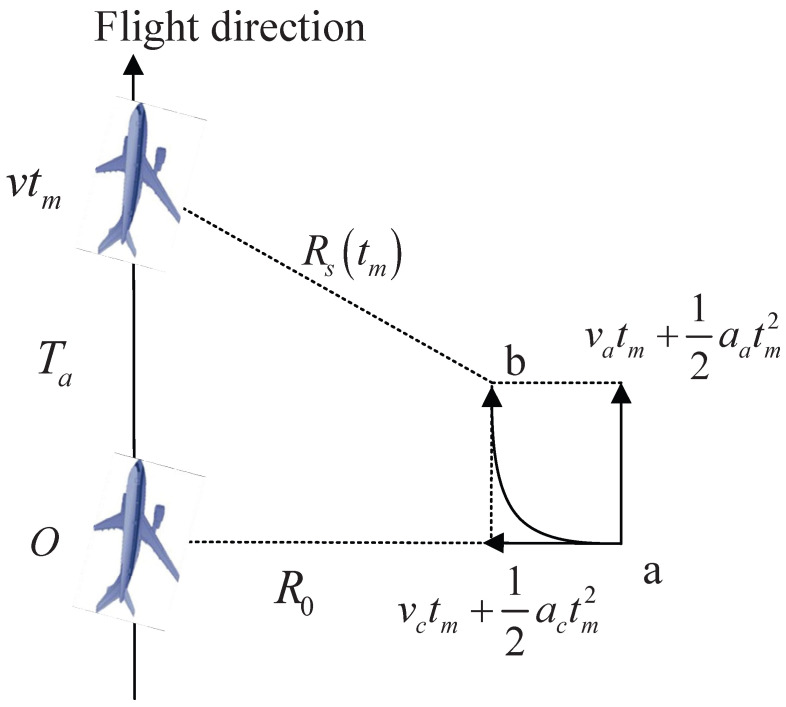
Geometric structure of an airborne radar sensor platform and a ground maneuvering target.

The geometric structure of an airborne radar sensor platform with velocity *v* and a ground maneuvering target is shown in Figure 1, which is defined in a 2-D slant plane with a side-looking work mode.

Assume that during the radar illumination time $T_{a}$, the along-track velocity $v_{a}$, the cross-track velocity $v_{c}$, the along-track acceleration $a_{a}$, and the cross-track acceleration $a_{c}$ are constant. $R_{0}$ and $R_{s}\left(t_{m}\right)$ denote the nearest and instantaneous slant range, respectively. According to Figure 1, $R_{s}\left(t_{m}\right)$ can be calculated as

(1) $$\begin{array}{cc} \; & R_{s}\left(t_{m}\right)\\ & =\sqrt{{\left(vt_{m}-v_{a}t_{m}-\frac{1}{2}a_{a}t_{m}^{2}\right)}^{2}+{\left(R_{0}-v_{c}t_{m}-\frac{1}{2}a_{c}t_{m}^{2}\right)}^{2}}, \end{array}$$

where $t_{m}$ is the slow-time variable.

In terms of the Taylor series expansion principle, under the condition of $\left(v-v_{a}\right)T_{a}\ll R_{0}$, $R_{s}\left(t_{m}\right)$ can be expanded to a third-order term, i.e.,

(2) $$R_{s}\left(t_{m}\right)\approx R_{0}+a_{1}t_{m}+a_{2}t_{m}^{2}+a_{3}t_{m}^{3},$$

where $a_{1}$, $a_{2}$, and $a_{3}$ represent the equivalent range velocity, acceleration, and jerk of $R_{s}\left(t_{m}\right)$, respectively, and the expressions of $a_{1}$, $a_{2}$, and $a_{3}$ are

(3) $$\begin{array}{cc} \; & a_{1}=-v_{c},\\ a_{2} & =\frac{{\left(v-v_{a}\right)}^{2}}{2R_{0}}-\frac{a_{c}}{2},\\ a_{3}= & \frac{v_{c}{\left(v-v_{a}\right)}^{2}}{2R_{0}^{2}}+\frac{a_{a}\left(v_{a}-v\right)}{2R_{0}}. \end{array}$$

Suppose that the radar transmits a narrow-band linear frequency-modulated (LFM) signal with the following form:

(4) $$s\left(t\right)=\mathrm{rect}\left(\frac{t}{T_{p}}\right)\exp\left(j\pi \mu t^{2}\right)\exp\left(j2\pi f_{c}t\right),$$

where

$$\mathrm{rect}\left(\frac{t}{T_{p}}\right)=\left\{\begin{array}{cc} 1, & |t|\leq \frac{t}{T_{p}}\\ 0, & |t|>\frac{t}{T_{p}} \end{array}\right.$$

μ is the chirp rate, $T_{p}$ denotes the pulse duration, $f_{c}$ is the carrier frequency, and *t* stands for the fast-time variable. Then, the received baseband signal can be stated as

(5) $$\begin{array}{cc} s_{r}\left(t,t_{m}\right) & =A_{0}\mathrm{rect}\left[\frac{t-2R_{s}\left(t_{m}\right)/c}{T_{p}}\right]\exp\left[-j4\pi \frac{R_{s}\left(t_{m}\right)}{\lambda }\right]\\ & \times \exp\left[j\pi \mu {\left(t-\frac{2R_{s}\left(t_{m}\right)}{c}\right)}^{2}\right], \end{array}$$

where $A_{0}$ denotes the target’s backscattering coefficient and *c* and $\lambda =c/f_{c}$ are the light speed and wavelength, respectively.

Bringing (2) into (5), the received signal of a maneuvering target after the pulse compression (PC) can be expressed as

(6) $$\begin{array}{cc} s_{1}\left(t,t_{m}\right) & =A_{1}\mathrm{sinc}\left[B\left(t-\frac{2(R_{0}+a_{1}t_{m}+a_{2}t_{m}^{2}+a_{3}t_{m}^{3})}{c}\right)\right]\\ & \times \exp\left[-j\frac{4\pi (R_{0}+a_{1}t_{m}+a_{2}t_{m}^{2}+a_{3}t_{m}^{3})}{\lambda }\right], \end{array}$$

where $A_{1}$ is the amplitude after PC. It can be observed from (6) that the sinc function characterizes the variation in the target’s envelope position, where the fast-time variable *t* is modulated by the slow-time variable $t_{m}$. When the position shift of the echo exceeds one range cell over the radar observation interval, envelope migrations, involving LRM, RC, and TRM, arise due to the equivalent range velocity, acceleration, and jerk. Additionally, the exponential term of (6) embodies a third-order phase function with respect to slow time. The second- and third-order terms in the phase function, i.e., $\exp\left(-j\frac{4\pi a_{2}t_{m}^{2}}{\lambda }\right)$ and $\exp\left(-j\frac{4\pi a_{3}t_{m}^{3}}{\lambda }\right)$, result in linear DFM (LDFM) and QDFM, respectively. These migration effects disperse the target energy in both range and Doppler domains, thereby complicating detection and parameter estimation.

## 3. Proposed Coherent Integration Algorithm

### 3.1. RM Compensation by RFRT

Performing the fast Fourier transform (FFT) on the fast-time variable *t* of (6), one has

(7) $$\begin{array}{cc} \; & S_{1}\left(f,t_{m}\right)\\ & =A_{2}\mathrm{rect}\left(\frac{f}{\mu T_{p}}\right)\exp\left[-j4\pi f\frac{\left(R_{0}+a_{1}t_{m}+a_{2}t_{m}^{2}+a_{3}t_{m}^{3}\right)}{c}\right]\\ & \times \exp\left[-j4\pi \frac{\left(R_{0}+a_{1}t_{m}+a_{2}t_{m}^{2}+a_{3}t_{m}^{3}\right)}{\lambda }\right], \end{array}$$

where $A_{2}$ is the amplitude after FFT along the fast-time dimension and *f* is the range frequency of *t*.

Upon examining the range frequency signal in (7), it can be observed that three coupled terms involving the range frequency variable *f* and the slow-time variable $t_{m}$ are present. Taking advantage of the symmetric equal-interval sampling of the range frequency variable, the coupling effects between range frequency and slow time can be effectively eliminated by constructing a range frequency reversal version of the echo signal and multiplying it with the original signal. This operation is referred to as the RFRT. The detailed processing steps are described below. Assume that $F_{s}$ is the sampling frequency; the discrete range frequency expression can be written as

(8) $$f=-\frac{F_{s}}{2},-\frac{F_{s}}{2}+\frac{F_{s}}{N},\ldots ,\frac{F_{s}}{2}-\frac{F_{s}}{N},\frac{F_{s}}{2},$$

where *N* is the total number of sampling points for range frequency.

By reversing $S_{1}\left(f,t_{m}\right)$ along the range frequency dimension, one can obtain the reversal signal $S_{1}\left(\overset{\leftarrow }{f},t_{m}\right)$,

(9) $$\begin{array}{cc} \; & S_{1}\left(\overset{\leftarrow }{f},t_{m}\right)\\ & =A_{2}\mathrm{rect}\left(\frac{\overset{\leftarrow }{f}}{\mu T_{p}}\right)\exp\left[-j4\pi \frac{\left(R_{0}+a_{1}t_{m}+a_{2}t_{m}^{2}+a_{3}t_{m}^{3}\right)}{\lambda }\right]\\ & \times \exp\left[-j4\pi \overset{\leftarrow }{f}\frac{\left(R_{0}+a_{1}t_{m}+a_{2}t_{m}^{2}+a_{3}t_{m}^{3}\right)}{c}\right], \end{array}$$

where the symbol “←” denotes the RFRT operation and the reversal range frequency can be expressed as

(10) $$\overset{\leftarrow }{f}=\frac{F_{s}}{2},\frac{F_{s}}{2}-\frac{F_{s}}{N},\ldots ,-\frac{F_{s}}{2}+\frac{F_{s}}{N},-\frac{F_{s}}{2}.$$

It can be observed from (8) and (10) that the range frequency *f* and the reversal range frequency $\overset{\leftarrow }{f}$ have the following relationship:

(11) $$f=-\overset{\leftarrow }{f},$$

which satisfies

(12) $$\begin{array}{c} \mathrm{rect}\left(\frac{f}{\mu T_{p}}\right)=\mathrm{rect}\left(\frac{\overset{\leftarrow }{f}}{\mu T_{p}}\right). \end{array}$$

Therefore, $S_{1}\left(\overset{\leftarrow }{f},t_{m}\right)$ can be rewritten as

(13) $$\begin{array}{cc} \; & S_{1}\left(\overset{\leftarrow }{f},t_{m}\right)=A_{2}\mathrm{rect}\left(\frac{f}{\mu T_{p}}\right)\\ & \exp\left[j4\pi f\frac{\left(R_{0}+a_{1}t_{m}+a_{2}t_{m}^{2}+a_{3}t_{m}^{3}\right)}{c}\right]\\ & \times \exp\left[-j4\pi \frac{\left(R_{0}+a_{1}t_{m}+a_{2}t_{m}^{2}+a_{3}t_{m}^{3}\right)}{\lambda }\right]. \end{array}$$

By multiplying (13) with (7), we can obtain the echo signal

(14) $$\begin{array}{cc} \; & S_{2}\left(f,t_{m}\right)=S_{1}\left(f,t_{m}\right)S_{1}\left(\overset{\leftarrow }{f},t_{m}\right)\\ & =A_{2}^{2}\mathrm{rec}t^{2}\left(\frac{f}{\mu T_{p}}\right)\\ & \times \exp\left[-j\frac{8\pi }{\lambda }\left(R_{0}+a_{1}t_{m}+a_{2}t_{m}^{2}+a_{3}t_{m}^{3}\right)\right]\\ & =A_{3}\mathrm{rec}t^{2}\left(\frac{f}{\mu T_{p}}\right)\exp\left[-j\frac{8\pi }{\lambda }\left(R_{0}+a_{1}t_{m}+a_{2}t_{m}^{2}+a_{3}t_{m}^{3}\right)\right], \end{array}$$

where $A_{3}=A_{2}^{2}$ denotes the amplitude. After the RFRT operation, the coupling effects of *f* and $t_{m}$ are removed, and thus RMs are compensated simultaneously, involving LRM, RC, and TRM. Performing the inverse FFT (IFFT) on (14) along the range frequency dimension, one has

(15) $$\begin{array}{cc} \; & s_{2}\left(t,t_{m}\right)=\mathrm{IFF}T_{f}\left[S_{2}\left(f,t_{m}\right)\right]\\ & =A_{4}\mathrm{sinc}\left[Bt\right]\exp\left[-j\frac{8\pi }{\lambda }\left(R_{0}+a_{1}t_{m}+a_{2}t_{m}^{2}+a_{3}t_{m}^{3}\right)\right], \end{array}$$

where $A_{4}$ represents the signal amplitude in the $t-t_{m}$ domain and $\mathrm{IFF}T_{f}\left[\cdot \right]$ denotes the IFFT operation on range frequency.

From (15), it is evident that the signal energy is concentrated in one range cell. However, the exponential terms indicate that DFMs, including LDFM and QDFM, still exist due to the equivalent range acceleration and jerk.

### 3.2. QDFM Correction by PCF

By extracting the signal from the target range cell, the resulting echo can be modeled as a quadratic frequency-modulated (QFM) signal, i.e.,

(16) $$s_{3}\left(t_{m}\right)=A_{5}\exp\left[-j\frac{8\pi }{\lambda }\left(R_{0}+a_{1}t_{m}+a_{2}t_{m}^{2}+a_{3}t_{m}^{3}\right)\right],$$

where $A_{5}=A_{4}\mathrm{sinc}\left[Bt\right]$.

To eliminate the QDFM, a compensation function related to the equivalent range jerk is constructed. The expression of PCF is defined as

(17) $$\begin{array}{c} H\left(t_{m},a_{3}^{\prime }\right)=\exp\left(j8\pi \frac{a_{3}^{\prime }t_{m}^{3}}{\lambda }\right), \end{array}$$

where $a_{3}^{\prime }$ represents the searching parameter of the third-order coefficient.

Multiplying (17) by (16) engenders

(18) $$\begin{array}{cc} s_{4}\left(t_{m},a_{3}^{\prime }\right) & =s_{3}\left(t_{m}\right)H\left(t_{m},a_{3}^{\prime }\right)\\ & =A_{5}\exp\left[-j\frac{8\pi }{\lambda }\left(R_{0}+a_{1}t_{m}+a_{2}t_{m}^{2}\right)\right]\\ & \times \exp\left[-j\frac{8\pi \left(a_{3}-a_{3}^{\prime }\right)t_{m}^{3}}{\lambda }\right]. \end{array}$$

If and only if the searching parameter of $a_{3}^{\prime }$ is matched with the target’s equivalent range jerk, i.e., $a_{3}^{\prime }=a_{3}$, the QDFM would be removed. Therefore, (18) can be rewritten as

(19) $$\begin{array}{cc} s_{4}\left(t_{m},a_{3}\right) & =s_{3}\left(t_{m}\right)H\left(t_{m},a_{3}^{\prime };a_{3}\right)\\ & =A_{5}\exp\left[-j\frac{8\pi }{\lambda }\left(R_{0}+a_{1}t_{m}+a_{2}t_{m}^{2}\right)\right]. \end{array}$$

As can be seen in (19), the QFM signal is degenerated into an LFM signal after the QDFM compensation, and thus the accumulation of the signal energy can be realized by time–frequency analysis methods.

### 3.3. Coherent Integration via LVD

Owing to its search-free nature and excellent performance in analyzing LFM signals, the time–frequency method LVD [33] can be applied to estimate the centroid frequency and chirp rate, as well as to achieve coherent integration. The detailed discussion is shown as follows.

Firstly, the parametric symmetric instantaneous autocorrelation function (PSIAF) of $s_{4}\left(t_{m},a_{3}\right)$ is constructed as

(20) $$\begin{array}{cc} R(t_{m},\tau ) & =s_{4}\left(t_{m}+\frac{\tau +\tau_{0}}{2},a_{3}\right)s_{4}^{*}\left(t_{m}-\frac{\tau +\tau_{0}}{2},a_{3}\right)\\ & ={\left|A_{5}\right|}^{2}\exp\left[-j\frac{8\pi a_{1}\tau_{0}}{\lambda }\right]\exp\left[-j\frac{8\pi a_{1}\tau }{\lambda }\right]\\ & \times \exp\left[-j\frac{16\pi a_{2}\left(\tau +\tau_{0}\right)t_{m}}{\lambda }\right], \end{array}$$

where τ is a lag-time variable and $\tau_{0}$ represents a time-delay constant related to a scaling operator. It is observed that τ and $\tau_{0}$ are coupled with each other. In order to remove the coupling relationship between them, a scaling transform is adopted in (20). The scaling operator is defined as

(21) $$\begin{array}{c} t_{m}=\frac{t_{m}^{\prime }}{h(\tau +\tau_{0})}, \end{array}$$

where *h* is a constant scaling factor. Substituting (21) into (20), the variables of τ and $t_{m}$ are decoupled,

(22) $$\begin{array}{cc} R(t_{m}^{\prime },\tau ) & =|A_{5}{|}^{2}\exp\left[-j\frac{8\pi a_{1}\tau_{0}}{\lambda }\right]\\ & \times \exp\left[-j\frac{8\pi a_{1}\tau }{\lambda }\right]\exp\left[-j\frac{16\pi a_{2}t_{m}^{\prime }}{h\lambda }\right], \end{array}$$

Conventionally, $\tau_{0}$ and *h* are set to 1 to obtain a desirable centroid frequency chirp rate (CFCR) representation. As can be seen in (22), the coupled relationship is eliminated through the scaling operator. Thus, LVD accumulation can be achieved by performing a 2-D FFT on (22),

(23) $$\begin{array}{cc} L(f_{t_{m}^{\prime }},f_{\tau }) & =A_{6}\exp\left[-j\frac{8\pi a_{1}}{\lambda }\right]\\ & \times \delta \left(f_{\tau }+\frac{4a_{1}}{\lambda }\right)\delta \left(f_{t_{m}^{\prime }}+\frac{8a_{2}}{\lambda }\right), \end{array}$$

where $A_{6}$ is the amplitude after LVD. As shown in (23), the LVD operation can realize the LDFM elimination as well as target energy accumulation.

### 3.4. Parameter Estimations of the Proposed Method

The parameter estimation performance of the proposed method relies on the coherent integration of LVD peaks. However, constrained by the radar’s PRF, Doppler ambiguity inevitably occurs. This causes the velocity estimates to fold, resulting in measurements that only represent the baseband velocity, rather than the true unambiguous velocity of the maneuvering target.

Suppose that $M_{amb1}$ presents the target’s Doppler ambiguity number for a maneuvering target; its equivalent range velocity may exceed the PRF, which can be written as

(24) $$\begin{array}{c} a_{1}=v_{0}+\frac{M_{amb1}f_{r}\lambda }{2}, \end{array}$$

where $f_{r}$ is the PRF and $v_{b}=\frac{f_{r}\lambda }{2}$ denotes the blind velocity. $v_{0}=mod\left[a_{1},v_{b}\right]$ is the baseband velocity of $a_{1}$, which satisfies $|v_{0}|<v_{b}/2$. Substituting (24) into (7), (7) can be rewritten as

(25) $$\begin{array}{cc} \; & S_{5}\left(f,t_{m}\right)=A_{2}\mathrm{rect}\left(\frac{f}{\mu T_{p}}\right)\\ & \times \exp\left[-j\frac{4\pi }{c}\left(f+f_{c}\right)\left(R_{0}+v_{0}t_{m}+a_{2}t_{m}^{2}+a_{3}t_{m}^{3}\right)\right]\\ & \times \exp\left[-j\frac{4\pi }{c}\left(f+f_{c}\right)\frac{M_{amb1}f_{r}\lambda }{2}t_{m}\right]. \end{array}$$

Performing the RFRT operation on (25), one can obtain

(26) $$\begin{array}{cc} \; & S_{6}\left(f,t_{m}\right)=A_{3}\mathrm{rec}t^{2}\left(\frac{f}{\mu T_{p}}\right)\\ & \times \exp\left[-j\frac{8\pi }{\lambda }\left(R_{0}+v_{0}t_{m}+a_{2}t_{m}^{2}+a_{3}t_{m}^{3}\right)\right]\\ & \times \exp\left[-j\frac{8\pi }{\lambda }\frac{M_{amb1}f_{r}\lambda }{2}t_{m}\right]. \end{array}$$

Consider that $\exp\left(-j4\pi M_{amb1}f_{r}t_{m}\right)=1$; thus (26) can be rewritten as

(27) $$\begin{array}{cc} \; & S_{6}\left(f,t_{m}\right)\\ & =A_{3}\mathrm{rec}t^{2}\left(\frac{f}{\mu T_{p}}\right)\exp\left[-j\frac{8\pi }{\lambda }\left(R_{0}+v_{0}t_{m}+a_{2}t_{m}^{2}+a_{3}t_{m}^{3}\right)\right]. \end{array}$$

The RFRT operation induces a doubling of the signal’s Doppler phase. Consequently, when $2v_{0}>v_{b}$, Doppler ambiguity can likewise occur. Analogously to Equation (24), the velocity $2v_{0}$ can be defined as

(28) $$\begin{array}{c} 2v_{0}=v_{1}+\frac{M_{amb2}f_{r}\lambda }{2}, \end{array}$$

where $M_{amb2}$ denotes the Doppler ambiguity number of $2v_{0}$. Substituting (28) into (27) yields

(29) $$\begin{array}{cc} \; & S_{6}\left(f,t_{m}\right)\\ & =A_{3}\mathrm{rec}t^{2}\left(\frac{f}{\mu T_{p}}\right)\exp\left[-j\frac{8\pi }{\lambda }\left(R_{0}+a_{2}t_{m}^{2}+a_{3}t_{m}^{3}\right)\right]\\ & \times \exp\left(-j\frac{4\pi }{\lambda }v_{1}t_{m}\right)\exp\left[-j\frac{4\pi }{\lambda }\frac{M_{amb2}f_{r}\lambda }{2}t_{m}\right]. \end{array}$$

By the same token, $\exp\left(-j2\pi M_{amb2}f_{r}t_{m}\right)=1$, and (29) can be simplified as follows

(30) $$\begin{array}{cc} \; & S_{6}\left(f,t_{m}\right)\\ & =A_{3}\mathrm{rec}t^{2}\left(\frac{f}{\mu T_{p}}\right)\exp\left[-j\frac{8\pi }{\lambda }\left(R_{0}+a_{2}t_{m}^{2}+a_{3}t_{m}^{3}\right)\right]\\ & \times \exp\left(-j\frac{4\pi }{\lambda }v_{1}t_{m}\right). \end{array}$$

Applying the IFFT along the range frequency dimension of (30) and extracting the signal at the target range cell yields the polynomial phase signal

(31) $$\begin{array}{cc} \; & S_{7}\left(t_{m}\right)\\ & =A_{5}\exp\left[-j\frac{8\pi }{\lambda }\left(R_{0}+a_{2}t_{m}^{2}+a_{3}t_{m}^{3}\right)\right]\exp\left(-j\frac{4\pi }{\lambda }v_{1}t_{m}\right). \end{array}$$

Thereafter, applying the PCF to remove QDFM and employing the LVD for coherent integration, the accumulated output of the LVD is obtained,

(32) $$\begin{array}{cc} \; & L\left(f_{t_{m}^{\prime }},f_{\tau }\right)=\mathrm{LVD}\left[S_{7}\left(t_{m}\right)H\left(t_{m},a_{3}^{\prime };a_{3}\right)\right]\\ & =A_{6}\exp\left[-j\frac{4\pi v_{1}}{\lambda }\right]\delta \left(f_{\tau }+\frac{2v_{1}}{\lambda }\right)\delta \left(f_{t_{m}^{\prime }}+\frac{8a_{2}}{\lambda }\right), \end{array}$$

where LVD[·] denotes the LVD transformation. The estimates for $v_{1}$ and $a_{2}$ are derived by locating the peak in the LVD domain and reading its 2D coordinates. According to (32), this peak position is given by

(33) $$\begin{array}{c} \left(f_{t_{m}^{\prime }},f_{\tau }\right)=\left(-\frac{8a_{2}}{\lambda },-\frac{2v_{1}}{\lambda }\right). \end{array}$$

Thus, based on the peak location, $\hat{v_{1}}$, $\hat{a_{2}}$, and $\hat{a_{3}}$ are estimated,

(34) $$\begin{array}{cc} \; & \hat{a_{3}}=\underset{a_{3}^{\prime }}{\arg\max}|\mathrm{LVD}\left[S_{7}\left(t_{m}\right)H\left(t_{m},a_{3}^{\prime }\right)\right]|,\\ \hat{v_{1}} & =-\frac{\lambda }{2}\times \left\{\underset{f_{\tau }}{\arg\max}|\mathrm{LVD}\left[S_{7}\left(t_{m}\right)H\left(t_{m},\hat{a_{3}}\right)\right]|\right\},\\ \hat{a_{2}} & =-\frac{\lambda }{8}\times \left\{\underset{f_{t_{m}^{\prime }}}{\arg\max}|\mathrm{LVD}\left[S_{7}\left(t_{m}\right)H\left(t_{m},\hat{a_{3}}\right)\right]|\right\}. \end{array}$$

In terms of the estimated parameters, the Doppler ambiguity numbers, i.e., $M_{amb1}$ and $M_{amb2}$, can be obtained by

(35) $$\begin{array}{c} \begin{array}{cc} \; & \left({\hat{M}}_{amb1},{\hat{M}}_{amb2}\right)=\\ & \underset{M_{amb1}^{\prime },M_{amb2}^{\prime }}{\arg\max}\left\{\mathrm{FF}T_{t_{m}}\left[\mathrm{IFF}T_{f}\left[S_{1}\left(f,t_{m}\right)H_{d}\left(M_{amb1}^{\prime },M_{amb2}^{\prime }\right)\right]\right]\right\}, \end{array} \end{array}$$

where the compensation function is

(36) $$\begin{array}{cc} \; & H_{d}\left(M_{amb1}^{\prime },M_{amb2}^{\prime }\right)\\ & =\exp\left(j\frac{2\pi }{c}\left(f+f_{c}\right)\left(\hat{v_{1}}+\frac{M_{amb2}^{\prime }f_{r}\lambda }{2}\right)t_{m}\right)\\ & \times \exp\left(j\frac{4\pi }{c}f\frac{M_{amb1}^{\prime }f_{r}\lambda }{2}t_{m}\right)\\ & \times \exp\left(j\frac{4\pi }{c}\left(f+f_{c}\right)\left(\hat{a_{2}}t_{m}^{2}+\hat{a_{3}}t_{m}^{3}\right)\right). \end{array}$$

According to the estimated Doppler ambiguity numbers and baseband velocity $v_{1}$, the equivalent velocity $a_{1}$ can be gained as follows:

(37) $$\begin{array}{c} {\hat{a}}_{1}=\frac{\hat{v_{1}}}{2}+\frac{{\hat{M}}_{amb2}f_{r}\lambda }{4}+\frac{{\hat{M}}_{amb1}f_{r}\lambda }{2}. \end{array}$$

Some important remarks should be noted:

**Remark** **1.** *In contrast to KT-based or RFT-based approaches, the proposed method employs the RFRT to achieve the RM compensation by directly decoupling slow-time and fast-frequency variables. This strategy circumvents the interference of ambiguous velocity and thus eliminates the detrimental effects of Doppler ambiguity and BSSL on target detection performance.*

**Remark** **2.** *Due to the fact that the Doppler phase satisfies an integer multiple of 2π, the polynomial phase signal after the LVD process can only obtain an estimate of the baseband velocity. Thus, the parameter estimation of the velocity should take into account the Doppler ambiguity number.*

**Remark** **3.** *To evaluate the actual velocity, it is necessary to estimate the Doppler ambiguity numbers. It is worth noting that $|v_{0}|<\frac{v_{b}}{2}$, and thus ${\hat{M}}_{amb2}$ usually takes values within {0, 1}.*

**Figure 2:**
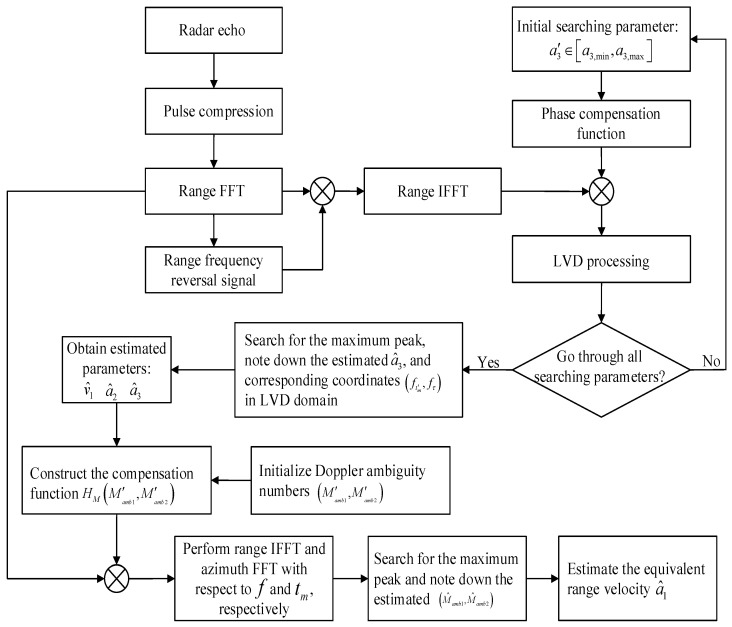
Detailed flowchart of the proposed method.

Next, the summarized procedures of the proposed target detection and parameter estimation method are given as follows, while the flowchart is shown in Figure 2.

Step 1: The radar transmits the LFM signal s(t) and receives the baseband signal $s_{r}\left(t,t_{m}\right)$.

Step 2: PC is performed on $s_{r}\left(t,t_{m}\right)$, and then the FFT operation is applied for *t* to obtain the compressed signal $S_{1}\left(f,t_{m}\right)$.

Step 3: Reversing $S_{1}\left(f,t_{m}\right)$ along the range frequency dimension can obtain the reversal signal $S_{1}\left(\overset{\leftarrow }{f},t_{m}\right)$. Thereafter, $S_{2}\left(f,t_{m}\right)$ is gained by implementing the RFRT operation via (14).

Step 4: The searching parameter $a_{3}^{\prime }\in \left[a_{3,min},a_{3,max}\right]$ is initialized and the phase compensation function $H(t_{m},a_{3}^{\prime })$ is constructed.

Step 5: IFFT is executed on $S_{2}\left(f,t_{m}\right)$ along the variable *f* and the target signal $s_{3}\left(t_{m}\right)$ is extracted. Each $a_{3}^{\prime }$ in $\left[a_{3,min},a_{3,max}\right]$ is examined, and the QDFM is compensated by (18).

Step 6: The LVD transform is performed on each searching parameter $a_{3}^{\prime }$ to obtain the LVD map $L(f_{t_{m}^{\prime }},f_{\tau })$.

Step 7: The maximal peak value is found, and then the corresponding motion parameters, i.e., equivalent jerk $\hat{a_{3}}$, baseband velocity $\hat{v_{1}}$, and equivalent acceleration $\hat{a_{2}}$, are estimated.

Step 8: The compensation function $H_{d}\left(M_{amb1}^{\prime },M_{amb2}^{\prime }\right)$ is constructed based on the three estimated parameters obtained in step 7 and multiplied by the received signal $S_{1}\left(f,t_{m}\right)$.

Step 9: According to the peak location, the estimated Doppler ambiguity numbers $\left({\hat{M}}_{amb1},{\hat{M}}_{amb2}\right)$ can be obtained. Afterwards, the equivalent velocity $\hat{a_{1}}$ can be calculated by (37).

## 4. Performance Analyses of the Proposed Method

### 4.1. Coherent Integration for Multiple Targets

Having established the methodology for motion compensation and parameter estimation of a single ground maneuvering target, we now consider a more realistic multi-target scenario. The nonlinear transforms integral to RFRT-SLVD can introduce cross-term interference in such environments. A subsequent analysis of the method’s focusing performance for multiple ground maneuvering targets is thus presented.

Assume that there are *J* maneuvering targets and the received signal of the *J* targets is written as

(38) $$\begin{array}{cc} s_{\mathrm{mult}}\left(t,t_{m}\right) & =\sum_{j=1}^{J}A_{j0}\mathrm{rect}\left[\frac{t-\tau_{j}}{T_{p}}\right]\exp\left[j\pi \mu {\left(t-\tau_{j}\right)}^{2}\right]\\ & \times \exp\left[-j2\pi f_{c}\tau_{j}\right], \end{array}$$

where $A_{j0}$ is the scattering coefficient of the *j*th target and $\tau_{j}$ is the propagation delay with complex motions,

(39) $$\begin{array}{c} \tau_{j}=\frac{2(R_{0j}+a_{1j}t_{m}+a_{2j}t_{m}^{2}+a_{3j}t_{m}^{3})}{c}, \end{array}$$

where $R_{0j}$ denotes the nearest slant range of the *j*th target and $a_{1j}$, $a_{2j}$, and $a_{3j}$ indicate the equivalent velocity, acceleration, and jerk of the *j*th target, respectively.

Substituting (39) into (38), the received signal of the *J* targets after PC could be expressed as

(40) $$\begin{array}{cc} \; & s_{\mathrm{mult},1}\left(t,t_{m}\right)=\\ & \sum_{j=1}^{J}A_{j1}\mathrm{sinc}\left[B\left(t-\frac{2(R_{0j}+a_{1j}t_{m}+a_{2j}t_{m}^{2}+a_{3j}t_{m}^{3})}{c}\right)\right]\\ & \times \exp\left[-j\frac{4\pi }{\lambda }\left(R_{0j}+a_{1j}t_{m}+a_{2j}t_{m}^{2}+a_{3j}t_{m}^{3}\right)\right], \end{array}$$

where $A_{j1}$ is the complex amplitude of the *j*th target after PC.

Performing the range FFT on (40) yields

(41) $$\begin{array}{cc} \; & S_{\mathrm{mult},1}\left(f,t_{m}\right)\\ & =\sum_{j=1}^{J}A_{j2}\mathrm{rect}\left(\frac{f}{\mu T_{p}}\right)\\ & \times \exp\left[-j\frac{4\pi }{c}f\left(R_{0j}+a_{1j}t_{m}+a_{2j}t_{m}^{2}+a_{3j}t_{m}^{3}\right)\right]\\ & \times \exp\left[-j\frac{4\pi }{\lambda }\left(R_{0j}+a_{1j}t_{m}+a_{2j}t_{m}^{2}+a_{3j}t_{m}^{3}\right)\right], \end{array}$$

where $A_{j2}$ denotes the complex amplitude of the *j*th target after the range FFT.

Then the reversal signal $S_{\mathrm{mult},1}\left(\overset{\leftarrow }{f},t_{m}\right)$ could be recast as

(42) $$\begin{array}{cc} \; & S_{\mathrm{mult},1}\left(\overset{\leftarrow }{f},t_{m}\right)\\ & =\sum_{j=1}^{J}A_{j2}\mathrm{rect}\left(\frac{\overset{\leftarrow }{f}}{\mu T_{p}}\right)\\ & \times \exp\left[-j\frac{4\pi }{c}f\left(R_{0j}+a_{1j}t_{m}+a_{2j}t_{m}^{2}+a_{3j}t_{m}^{3}\right)\right]\\ & \times \exp\left[-j\frac{4\pi }{\lambda }\left(R_{0j}+a_{1j}t_{m}+a_{2j}t_{m}^{2}+a_{3j}t_{m}^{3}\right)\right]. \end{array}$$

Applying the RFRT operation, one has

(43) $$\begin{array}{cc} S_{\mathrm{mult},\mathrm{RFRT}}\left(f,t_{m}\right) & =S_{\mathrm{mult},1}\left(f,t_{m}\right)S_{\mathrm{mult},1}\left(\overset{\leftarrow }{f},t_{m}\right)\\ & =S_{\mathrm{mult},\mathrm{auto}}\left(f,t_{m}\right)+S_{\mathrm{mult},\mathrm{cross}}\left(f,t_{m}\right), \end{array}$$

where $S_{\mathrm{mult},\mathrm{auto}}\left(f,t_{m}\right)$ and $S_{\mathrm{mult},\mathrm{cross}}\left(f,t_{m}\right)$, respectively, denote the auto terms and cross terms, whose expressions can be given by (44) and (45),

(44) $$\begin{array}{cc} \; & S_{\mathrm{mult},\mathrm{auto}}\left(f,t_{m}\right)\\ & =\sum_{j=1}^{J}A_{j2}^{2}\mathrm{rec}t^{2}\left(\frac{f}{\mu T_{p}}\right)\\ & \times \exp\left[-j\frac{8\pi }{\lambda }\left(R_{0j}+a_{1j}t_{m}+a_{2j}t_{m}^{2}+a_{3j}t_{m}^{3}\right)\right], \end{array}$$

(45) $$\begin{array}{cc} \; & S_{\mathrm{mult},\mathrm{cross}}\left(f,t_{m}\right)\\ & =\sum_{i,j=1,i\neq j}^{J}A_{i2}A_{j2}\mathrm{rec}t^{2}\left(\frac{f}{\mu T_{p}}\right)\\ & \times \exp\{-j\frac{4\pi }{c}f[\left(R_{0i}-R_{0j}\right)+\left(a_{1i}-a_{1j}\right)t_{m}\\ & \;\;\;+\left(a_{2i}-a_{2j}\right)t_{m}^{2}+\left(a_{3i}-a_{3j}\right)t_{m}^{3}]\}\\ & \times \exp\{-j\frac{4\pi }{\lambda }[\left(R_{0i}+R_{0j}\right)+\left(a_{1i}+a_{1j}\right)t_{m}\\ & \;\;\;+\left(a_{2i}+a_{2j}\right)t_{m}^{2}+\left(a_{3i}+a_{3j}\right)t_{m}^{3}]\}. \end{array}$$

After performing the range IFFT on (43) along variable *f*, we have

(46) $$\begin{array}{cc} s_{\mathrm{mult},\mathrm{RFRT}}\left(t,t_{m}\right) & =\mathrm{IFF}T_{f}\left[S_{\mathrm{mult},\mathrm{RFRT}}\left(f,t_{m}\right)\right]\\ & =s_{\mathrm{mult},\mathrm{auto}}\left(t,t_{m}\right)+s_{\mathrm{mult},\mathrm{cross}}\left(t,t_{m}\right), \end{array}$$

where $s_{\mathrm{mult},\mathrm{auto}}\left(t,t_{m}\right)$ and $s_{\mathrm{mult},\mathrm{cross}}\left(t,t_{m}\right)$ represent the auto terms and the cross terms in the $t-t_{m}$ domain, respectively, whose expressions are shown in (47) and (48),

(47) $$\begin{array}{cc} \; & s_{\mathrm{mult},\mathrm{auto}}\left(t,t_{m}\right)\\ & =\sum_{j=1}^{J}A_{jj3}\mathrm{sinc}\left(Bt\right)\\ & \times \exp\left[-j\frac{8\pi }{\lambda }\left(R_{0j}+a_{1j}t_{m}+a_{2j}t_{m}^{2}+a_{3j}t_{m}^{3}\right)\right], \end{array}$$

(48) $$\begin{array}{cc} \; & s_{\mathrm{mult},\mathrm{cross}}\left(t,t_{m}\right)\\ & =\sum_{i,j=1,i\neq j}^{J}A_{ij3}\\ & \;\times \mathrm{sinc}\{B[t-\frac{2}{c}(\left(R_{0i}-R_{0j}\right)+\left(a_{1i}-a_{1j}\right)t_{m}\\ & \;\;\;+\left(a_{2i}-a_{2j}\right)t_{m}^{2}+\left(a_{3i}-a_{3j}\right)t_{m}^{3})]\}\\ & \;\times \exp\{-j\frac{4\pi }{\lambda }[\left(R_{0i}+R_{0j}\right)+\left(a_{1i}+a_{1j}\right)t_{m}\\ & \;\;\;+\left(a_{2i}+a_{2j}\right)t_{m}^{2}+\left(a_{3i}+a_{3j}\right)t_{m}^{3}]\}, \end{array}$$

where $A_{jj3}$ is the auto term’s signal amplitude of the *j*th target after the IFFT operation and $A_{ij3}$ is the cross term’s signal amplitude between the *i*th target and the *j*th target.

From the above derivations, it can be seen that after the RFRT operation, the trajectories of auto terms are concentrated at the same range cell along t=0, which are parallel to the slow-time axis $t_{m}$. Meanwhile the peak value of the cross terms would distribute along the curved lines $t=\frac{2}{c}\left[\left(R_{0i}-R_{0j}\right)+\left(a_{1i}-a_{1j}\right)t_{m}+\left(a_{2i}-a_{2j}\right)t_{m}^{2}+\left(a_{3i}-a_{3j}\right)t_{m}^{3}\right]$, i,j=1, i≠j. In view of this feature, multiple target detection can be analyzed in three cases.

Case 1: When the nearest slant ranges of multiple targets are not equal, e.g., $\left(R_{0i}\;\neq \;R_{0j}\right)$, and meanwhile their equivalent motion parameters satisfy the conditions of $a_{1i}\;\neq \;a_{1j}$, $a_{2i}\;\neq \;a_{2j}$, or $a_{3i}\;\neq \;a_{3j}$(∃i,j=1,2,…,J,i≠j), the RMs of *J* auto terms could be completely removed and their trajectories would be parallel to the slow-time axis at the same range cell along t=0. In contrast, because of the different motion parameters, the RMs of cross terms still exist after the RFRT operation, and the signal envelopes change with the slow time. In this circumstance, the energies of cross terms could not undergo the subsequent accumulations. In addition, the trajectories of cross terms lie in different range cells. Therefore, one can fully distinguish the auto terms and the cross terms via range positions and eliminate the cross terms by extracting the trajectories along the range axis t=0.

Case 2: When the nearest slant ranges of some targets are equal $\left(R_{0i}=R_{0j}\right)$, and the equivalent motion parameters are different, i.e., $a_{1i}\;\neq \;a_{1j}$, $a_{2i}\;\neq \;a_{2j}$, or $a_{3i}\;\neq \;a_{3j}$(∃i,j=1,2,…,J,i≠j), the auto terms and cross terms can be distinguished by judging whether the trajectory is parallel to the slow-time axis. To illustrate, the analyses of two targets are shown. In this case, the *i*th and the *j*th auto terms can be, respectively, written as

(49) $$\begin{array}{cc} \; & s_{i,\mathrm{auto}}\left(t,t_{m}\right)=A_{ii3}\mathrm{sinc}\left(Bt\right)\\ & \times \exp\left[-j\frac{8\pi }{\lambda }\left(R_{0i}+a_{1i}t_{m}+a_{2i}t_{m}^{2}+a_{3i}t_{m}^{3}\right)\right], \end{array}$$

and

(50) $$\begin{array}{cc} \; & s_{j,\mathrm{auto}}\left(t,t_{m}\right)=A_{jj3}\mathrm{sinc}\left(Bt\right)\\ & \times \exp\left[-j\frac{8\pi }{\lambda }\left(R_{0j}+a_{1j}t_{m}+a_{2j}t_{m}^{2}+a_{3j}t_{m}^{3}\right)\right]. \end{array}$$

The cross-term expressions between the *i*th target and the *j*th target after the RFRT operation are expressed as

(51) $$\begin{array}{cc} \; & s_{ij,\mathrm{cross}}\left(t,t_{m}\right)\\ & =A_{ij3}\mathrm{sinc}\{B[t-\frac{2}{c}(\left(a_{1i}-a_{1j}\right)t_{m}\\ & \;\;\;+\left(a_{2i}-a_{2j}\right)t_{m}^{2}+\left(a_{3i}-a_{3j}\right)t_{m}^{3})]\}\\ & \;\times \exp\{-j\frac{4\pi }{\lambda }[\left(R_{0i}+R_{0j}\right)+\left(a_{1i}+a_{1j}\right)t_{m}\\ & \;\;\;+\left(a_{2i}+a_{2j}\right)t_{m}^{2}+\left(a_{3i}+a_{3j}\right)t_{m}^{3}]\}, \end{array}$$

(52) $$\begin{array}{cc} \; & s_{ji,\mathrm{cross}}\left(t,t_{m}\right)\\ & =A_{ji3}\mathrm{sinc}\{B[t-\frac{2}{c}(\left(a_{1j}-a_{1i}\right)t_{m}\\ & \;\;\;+\left(a_{2j}-a_{2i}\right)t_{m}^{2}+\left(a_{3j}-a_{3i}\right)t_{m}^{3})]\}\\ & \;\times \exp\{-j\frac{4\pi }{\lambda }[\left(R_{0j}+R_{0i}\right)+\left(a_{1j}+a_{1i}\right)t_{m}\\ & \;\;\;+\left(a_{2j}+a_{2i}\right)t_{m}^{2}+\left(a_{3j}+a_{3i}\right)t_{m}^{3}]\}. \end{array}$$

It can be seen from the above equations that although the center point of the cross-term trajectories is located in the same range cell t=0 as that of the auto terms, the tracks of cross terms are distributed along the curve $t=\pm \{\frac{2}{c}\left[\left(a_{1i}-a_{1j}\right)t_{m}+\left(a_{2i}-a_{2j}\right)t_{m}^{2}+\left(a_{3i}-a_{3j}\right)t_{m}^{3}\right]\}$. Thus, the energy of cross terms is still defocused due to the uncompensated RMs and they could not affect the detection of auto terms.

Case 3: Taking the two targets as examples, when the nearest slant ranges of some targets are different, but their equivalent motion parameters are equal, i.e., $R_{0i}\;\neq \;R_{0j}$, $a_{1i}=a_{1j}$, $a_{2i}=a_{2j}$, and $a_{3i}=a_{3j}$ (∃i,j=1,2,…,J,i≠j), the cross terms of the *i*th target and the *j*th target can be written as

(53) $$\begin{array}{cc} \; & s_{ij,\mathrm{cross}}\left(t,t_{m}\right)\\ & =A_{i3}A_{j3}\mathrm{sinc}\left\{B\left[t-\frac{2}{c}\left(R_{0i}-R_{0j}\right)\right]\right\}\\ & \;\times \exp\{-j\frac{4\pi }{\lambda }[\left(R_{0i}+R_{0j}\right)+\left(a_{1i}+a_{1j}\right)t_{m}\\ & \;\;\;+\left(a_{2i}+a_{2j}\right)t_{m}^{2}+\left(a_{3i}+a_{3j}\right)t_{m}^{3}]\}, \end{array}$$

(54) $$\begin{array}{cc} \; & s_{ji,\mathrm{cross}}\left(t,t_{m}\right)\\ & =A_{j3}A_{i3}\mathrm{sinc}\left\{B\left[t-\frac{2}{c}\left(R_{0j}-R_{0i}\right)\right]\right\}\\ & \;\times \exp\{-j\frac{4\pi }{\lambda }[\left(R_{0j}+R_{0i}\right)+\left(a_{1j}+a_{1i}\right)t_{m}\\ & \;\;\;+\left(a_{2j}+a_{2i}\right)t_{m}^{2}+\left(a_{3j}+a_{3i}\right)t_{m}^{3}]\}. \end{array}$$

As can be seen, the RMs of the cross terms are completely eliminated due to the same motion parameters of the two targets. And the trajectories of the two cross terms peak along $t=2(R_{0i}-R_{0j})/c$ and $t=2(R_{0j}-R_{0i})/c$, respectively, which are parallel to the slow-time axis. Moreover, we can observe that the two trajectories of cross terms are symmetrically distributed with respect to their auto terms and appear pairwise. In this case, the target signal can be obtained by taking the trajectories along the range axis t=0.

Synthesizing the above analyses, the RFRT operation shows that it distinguishes the auto terms and cross terms through extracting the tracks along the range cell t=0, while the subsequent LVD transform has a good cross-term suppression ability [33]. Therefore, the proposed algorithm can effectively suppress the cross terms and achieve the coherent integration as well as parameter estimation of the multiple targets.

### 4.2. Comparisons of Computational Complexity

In this section, we compare the computational complexities of the proposed method with the GRFT, the TRT-SKT-LVD, and ACCF iteratively. Suppose that the numbers of range cells and azimuth pulses are *N* and *M*, respectively. $N_{a_{1}}$, $N_{a_{2}}$, and $N_{a_{3}}$ denote the searching numbers of equivalent range velocity, acceleration, and jerk, respectively. $N_{\tau }$ and $N_{am}$ represent the amount of lag time to LVD and searching number for Doppler ambiguity.

For the proposed method RFRT-SLVD, the implementation procedures mainly include an N×M point multiplication operation, one-dimensional (1-D) parameter searching of jerk, LVD operation $O(5N_{\tau }M\log_{2}M+N_{\tau }M\log_{2}N_{\tau })$, and Doppler ambiguity number searching. Thus, the computational burden is

(55) $$\begin{array}{cc} C_{\mathrm{RFRT}\text{-}\mathrm{SLVD}}=O & [NM+N_{a_{3}}\left(5N_{\tau }M\log_{2}M+N_{\tau }M\log_{2}N_{\tau }\right)\\ & +2N_{am}\left(NM\log_{2}M+MN\log_{2}N\right)]. \end{array}$$

Similarly to the proposed algorithm, TRT-SKT-LVD also needs to consider the searching number of the Doppler ambiguity. The computational complexities of the TRT operation and SKT are O(NM) and $O(MN^{2}+NM\log_{2}N)$, respectively. Therefore, its computational load is

(56) $$\begin{array}{cc} C_{\mathrm{TRT}\text{-}\mathrm{SKT}\text{-}\mathrm{LVD}}=O & [NM+MN^{2}+NM\log_{2}N\\ & +N_{am}N\left(5N_{\tau }M\log_{2}M+N_{\tau }M\log_{2}N_{\tau }\right)]. \end{array}$$

For the GRFT method, it requires a 4-D parameter search with the computational complexity

(57) $$\begin{array}{c} C_{\mathrm{GRFT}}=O\left[N_{a_{1}}N_{a_{2}}N_{a_{3}}NM+N_{a_{1}}N_{a_{2}}N_{a_{3}}N\left(M-1\right)\right]. \end{array}$$

Compared with the above three algorithms, the main implementation procedures of the ACCF iteratively method only need multiplication operations, which is computationally efficient, with a computational cost of

(58) $$\begin{array}{c} C_{\mathrm{ACCF}\;\mathrm{iteratively}}=O\left[3NM\log_{2}N+3NM\log_{2}M+4MN\right]. \end{array}$$

**Figure 3:**
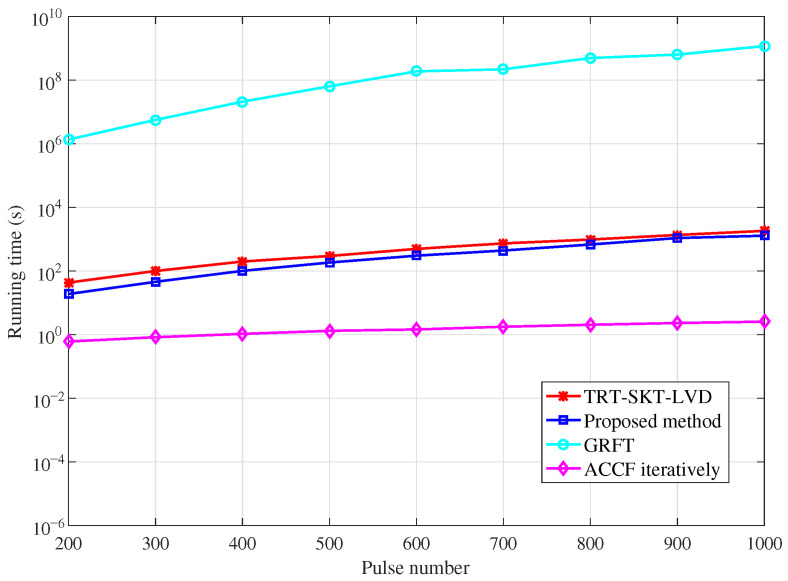
The comparisons of running time.

The computational complexity of the four algorithms is summarized in Table 1. Notably, the proposed algorithm requires only two 1-D searches, i.e., the equivalent range jerk and the Doppler ambiguity number, to accomplish target detection and parameter estimation, respectively. This structure results in a significantly lower computational cost compared to the GRFT method. Although the TRT-SKT-LVD method also remains efficient, its computational load is slightly higher than that of our proposed algorithm due to the need for a 2-D search over range cells and Doppler ambiguity numbers. Among all, the ACCF iterative algorithm achieves the lowest complexity by eliminating search operations entirely. It should be noted, however, that this computational efficiency comes at the expense of detection performance. To further support this analysis, we compare the running times of RFRT-SLVD, TRT-SKT-LVD, the GRFT, and ACCF iteratively via numerical simulations, with the number of pulses varying from 200 to 1000. The corresponding results are presented in Figure 3.

The computational complexities of RFRT-SLVD, TRT-SKT-LVD, the GRFT, and ACCF iteratively are compared via numerical simulation experiments. The value range of pulse numbers is from 200 to 1000. The comparisons of running time for the four methods are illustrated in Figure 3.

As can be seen, the running time of RFRT-SLVD is lower than those of TRT-SKT-LVD and the GRFT because the proposed method reduces searching dimensions and avoids an interpolation operation. Compared with the above three algorithms, the ACCF iteratively method has the lowest running time. However, the high efficiency of ACCF iteratively sacrifices accumulation performance.

## 5. Results and Discussion

This section presents a series of experiments to validate the effectiveness of the proposed algorithm, comprising single-target energy integration, multi-target scenario simulations, analyses of parameter estimation accuracy, and detection performance. The parameters of the radar system are shown in Table 2.

### 5.1. Coherent Integration for Single Target

In this section, we first conduct experiments on the coherent accumulation of a ground maneuvering target. Then, comparative experiments for a single target with several popular methods, i.e., TRT-SKT-LVD, ACCF iteratively, and the GRFT, are presented. The motion parameters of a maneuvering target are shown in Table 3.

**Figure 4:**
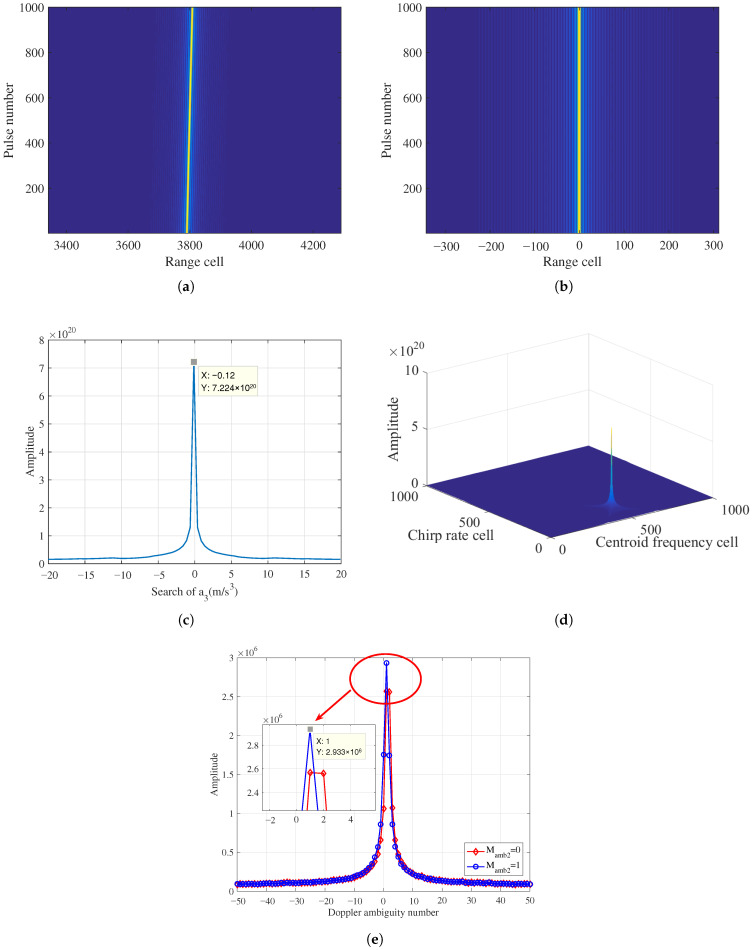
Simulation results of a ground maneuvering target: (**a**) Received signal after PC. (**b**) RM removal after RFRT. (**c**) The searching result of equivalent range jerk. (**d**) Coherent integration result of RFRT-SLVD. (**e**) The search of Doppler ambiguity numbers.

In terms of (3), the values of equivalent range velocity, acceleration, and jerk can be obtained as 45m/s, $2.5250\;m/s^{2}$, and $-0.1231\;m/s^{3}$, respectively. Simulation results of a ground maneuvering target without noise are shown in Figure 4.

Figure 4a gives the simulation result after PC, which indicates that the signal trajectory is subject to severe RMs caused by complex motions. Following the RFRT operation, Figure 4b shows that the RM effects have been effectively compensated, and the target trajectory returns to the same range cell, which is parallel to the slow-time axis.

Figure 4c displays the search result for the equivalent range jerk via PCF in (17). According to the peak value, we can easily find that the estimated equivalent range jerk is $-0.12\;m/s^{3}$. By substituting this estimated value into (17) and implementing Equations (18)–(23), the coherent accumulation result after the LVD transform can be obtained, as shown in Figure 4d, from which we can find that the energy is focused and integrated well.

Based on the peak position in Figure 4d, the estimated baseband velocity and equivalent range acceleration are calculated as −0.0075m/s and $2.5263\;m/s^{2}$, respectively. Figure 4e reveals the joint search results for the Doppler ambiguity numbers $M_{amb1}$ and $M_{amb2}$. Through simulation results, the values of Doppler ambiguity numbers can be determined as ${\hat{M}}_{amb1}=1$ and ${\hat{M}}_{amb2}=1$, respectively. Accordingly, it is possible to work out the estimated equivalent range velocity as 44.9962m/s.

**Figure 5:**
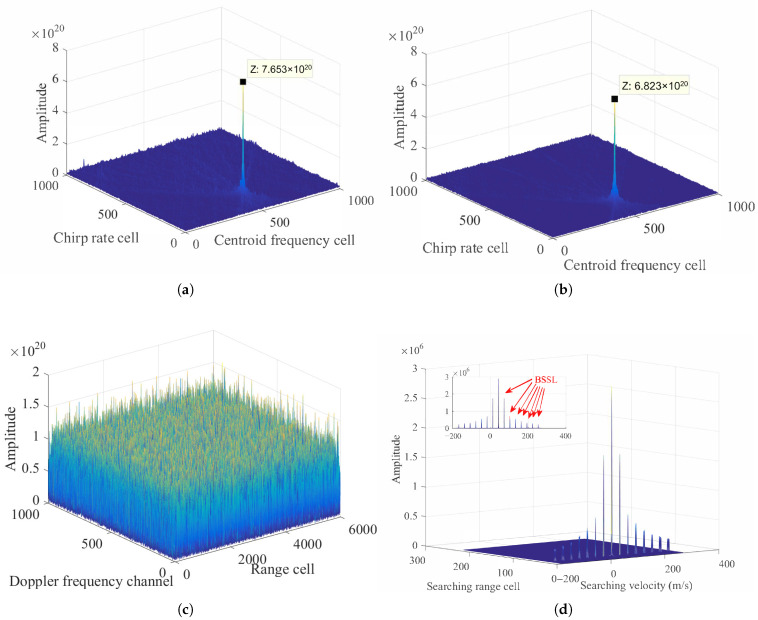
Coherent integration results for four methods: (**a**) RFRT-SLVD. (**b**) TRT-SKT-LVD. (**c**) ACCF iteratively. (**d**) GRFT.

For comparisons, the coherent integration results of RFRT-SLVD, TRT-SKT-LVD, ACCF iteratively, and the GRFT are illustrated in Figure 5. The SNR after PC is set as 6 dB.

Figure 5a and Figure 5b exhibit the coherent integration results of RFRT-SLVD and TRT-SKT-LVD, respectively. It can be seen that both methods can eliminate the RMs and DFMs and achieve coherent accumulation. However, the computational complexity of TRT-SKT-LVD is higher than that of RFRT-SLVD, owing to its requirement for a 2-D parameter search and the interpolation operation involved in the SKT. The accumulation result for ACCF iteratively is shown in Figure 5c. Compared with the proposed method, the ACCF approach exhibits difficulty in detecting the peak value due to its lower noise margin. Figure 5d displays the GRFT result. As illustrated, while the GRFT achieves coherent integration, it is plagued by the BSSL problem, which adversely affects target detection.

### 5.2. Analyses of Multiple Targets

To illustrate the cross-term suppression ability of the proposed algorithm, this section gives the analyses of multiple targets. In this example, we adopt two targets to perform the simulation experiments. The motion parameters of target 1 and target 2 are listed in Table 4.

As for setting parameters, the equivalent range velocity, acceleration, and jerk of target 1 can be calculated as 45m/s, $3.5333\;m/s^{2}$, and $-0.3043\;m/s^{3}$, respectively. While the equivalent range velocity, acceleration, and jerk of target 2 are −48m/s, $3.5417\;m/s^{2}$, and $0.5225\;m/s^{3}$, respectively.

**Figure 6:**
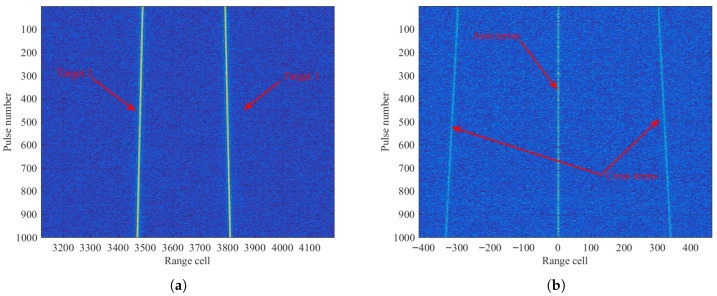

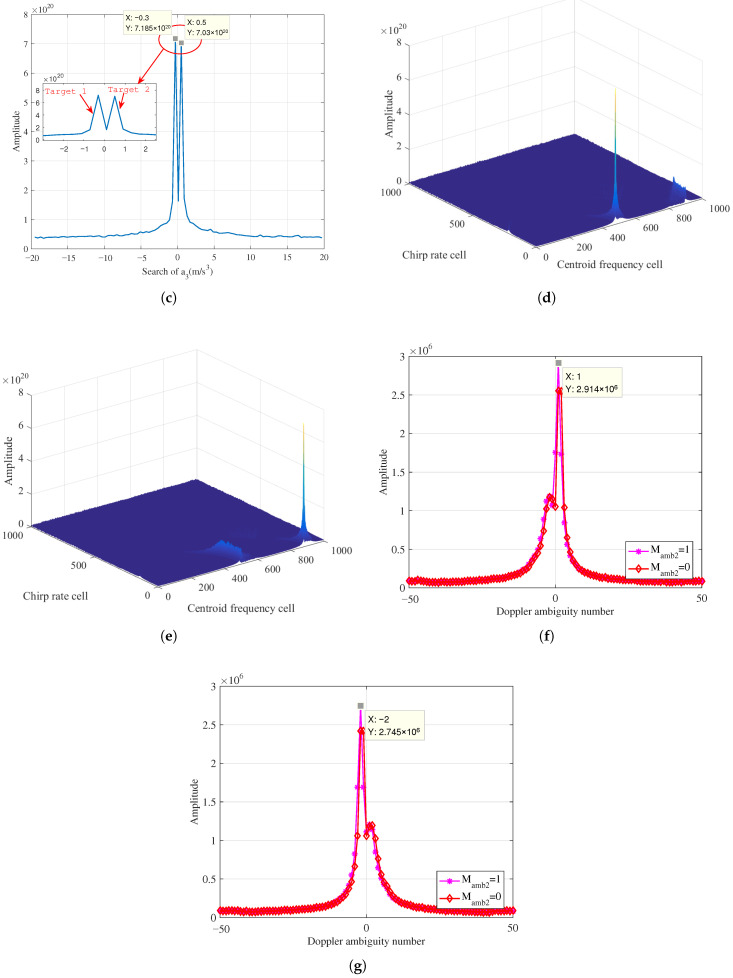
Simulation results of multiple targets: (**a**) Received signals after PC. (**b**) RM removal after RFRT. (**c**) The searching results of equivalent range jerk. (**d**) Coherent integration result of target 1. (**e**) Coherent integration result of target 2. (**f**) Doppler ambiguity numbers of target 1. (**g**) Doppler ambiguity numbers of target 2.

The experiment results of the multiple targets are shown in Figure 6. Figure 6a depicts the motion trajectories of target 1 and target 2 after PC in a noise environment, where both targets exhibit phenomena of complex RMs. The results after applying the RFRT operation are presented in Figure 6b. The observation states that after the RFRT operation, the auto terms of both targets are aligned within the same range cell t=0 and run parallel to the slow-time axis. In contrast, the cross terms of the two targets are symmetrically distributed on both sides of the auto terms, and their trajectories remain affected by RMs. Two conclusions can be drawn: (1) By extracting the auto terms at the fast time t=0, one can obtain the desired target signals and remove the cross terms. (2) The failure to effectively focus the energy of cross terms, due to their persistent RMs, results in their further suppression in the integration output.

The search results of the equivalent range jerk are depicted in Figure 6c. From the peak positions, the estimated equivalent range jerk of target 1 and 2 is $-0.3\;m/s^{3}$ and $0.5\;m/s^{3}$, respectively. Furthermore, the coherent integration results obtained via the LVD transform are shown in Figure 6d and Figure 6e, corresponding to target 1 and target 2, respectively. We can see that the energy of both targets is well focused. Based on the peak values, the estimated baseband velocity and equivalent range acceleration of target 1 are −0.0075m/s and $3.5323\;m/s^{2}$, respectively. Similarly, the corresponding estimates for target 2 can be calculated as −6.0135m/s and $3.5398\;m/s^{2}$, respectively. It should be noted that if the scattering intensities of different targets differ significantly, the CLEAN technique [34] can be employed to achieve coherent integration.

Figure 6f,g illustrate the joint search results for the Doppler ambiguity numbers. Specifically, for target 1, the estimated values are ${\hat{M}}_{amb1}=1$ and ${\hat{M}}_{amb2}=1$; for target 2, the estimates are ${\hat{M}}_{amb1}=-2$ and ${\hat{M}}_{amb2}=1$. According to the estimated Doppler ambiguity numbers, the equivalent range velocities of target 1 and target 2 are derived as 44.9962m/s and −48.0068m/s, respectively, which agree well with the theoretical values. A comparison between the preset parameters and the estimated motion parameters for this example is summarized in Table 5.

### 5.3. Parameter Estimation Performance

This section compares the parameter estimation performance of the three algorithms, i.e., RFRT-SLVD, the GRFT, and ACCF iteratively.

The motion parameters of the target are set as follows: the equivalent range velocity is 75m/s, the equivalent range acceleration is $2\;m/s^{2}$, and the equivalent range jerk is $1\;m/s^{3}$. The variation range of the SNR (after PC) is taken as [−20 dB:2 dB:20 dB]. The radar system parameters are the same as those listed in Table 2.

**Figure 7:**
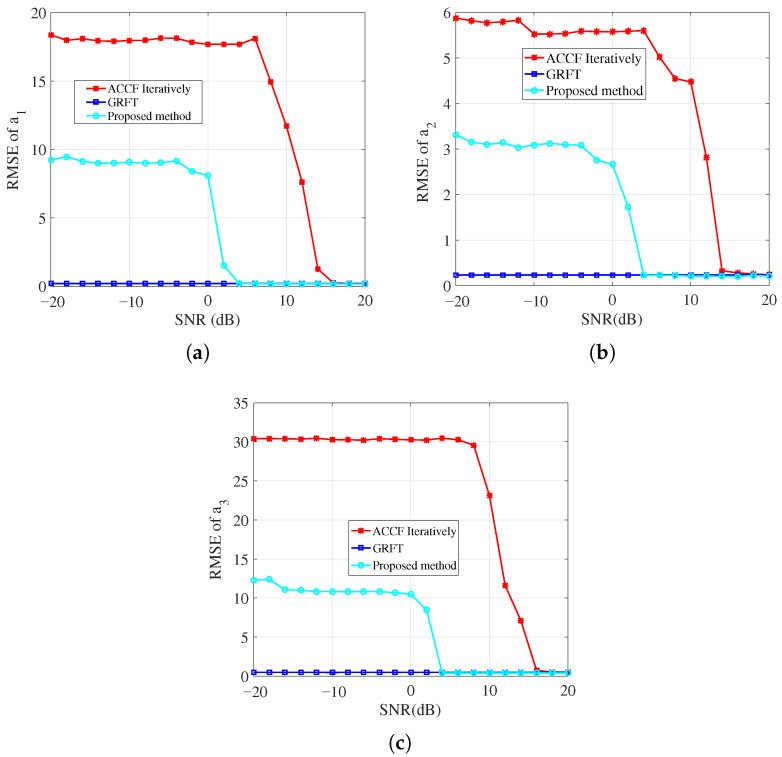
The parameter estimation performance of three methods: (**a**) The RMSE of equivalent range velocity. (**b**) The RMSE of equivalent range acceleration. (**c**) The RMSE of equivalent range jerk.

For each SNR level, 100 Monte Carlo trials were conducted. The resulting root mean square errors (RMSEs) of the three motion parameters versus the SNR are presented in Figure 7. Specifically. Figure 7a, Figure 7b and Figure 7c illustrate the RMSE curves for equivalent range velocity, acceleration, and jerk, respectively.

As can be seen from the figures, the GRFT yields superior parameter estimation accuracy compared to both RFRT-SLVD and ACCF iteratively. The SNR threshold for the proposed method is approximately 4 dB. When the input SNR exceeds this threshold, the estimation performance of the proposed algorithm approaches that of the GRFT method. Although both the proposed algorithm and ACCF iteratively method realize coherent integration via two complex multiplications, the proposed algorithm exhibits enhanced noise robustness due to the introduction of a 1-D lag-time variable. Consequently, the ACCF iteratively algorithm demonstrates inferior motion parameter estimation performance relative to the proposed technique.

### 5.4. Analyses of Detection Ability

The detection performance of the proposed algorithm is evaluated against five other algorithms (MTD, the RFT, ACCF iteratively, TRT-SKT-LVD, and the GRFT) through 1000 Monte Carlo simulations. The experiments are conducted under Gaussian noise conditions, with the input SNR after PC ranging from −30 dB to 40 dB in 2 dB increments. The target motion parameters and radar system parameters remain consistent with those used in the simulation of Figure 4, and a constant false alarm rate of $P_{f_{a}}=10^{-6}$ is maintained.

**Figure 8:**
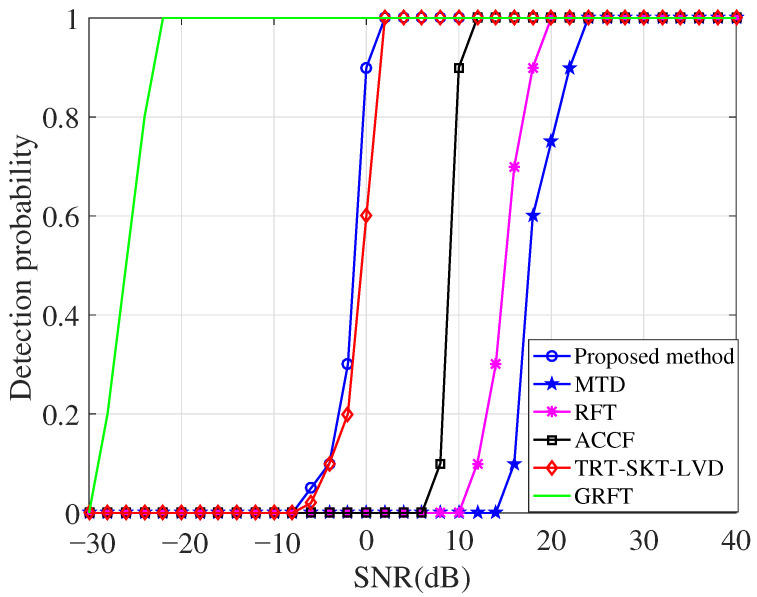
Detection probability of the six methods.

The detection probability curves are shown in Figure 8. It is observed that the MTD and RFT algorithms suffer from a severe integration performance loss due to the uncompensated high-order phase errors. In contrast, the remaining four algorithms can effectively compensate the RMs and DFMs, thus realizing the signal energy accumulation of a higher-order maneuvering target. Among them, the GRFT algorithm delivers superior detection performance relative to those of other three algorithms (ACCF iteratively, TRT-SKT-LVD, and the proposed algorithm). This advantage, however, comes at the cost of high computational complexity resulting from its required 3-D parameter search. The proposed algorithm reduces this burden substantially by condensing the search process from 3-D search to 1-D search. Although the TRT-SKT-LVD algorithm has a close detection performance to that of the proposed technique, it still relies on a computationally more expensive 2-D search. Furthermore, the proposed method outperforms the ACCF iteratively approach, which can be attributed to the additional coherent integration gain provided by the introduced 1-D lag-time variable. In summary, the proposed algorithm offers a favorable trade-off between detection performance and computational efficiency.

## 6. Conclusions

In this paper, a novel method of long-time coherent integration and high-order motion parameter estimation for a ground maneuvering target, namely RFRT-SLVD, is proposed. It settles the problems with the complex RMs and DFMs jointly caused by the equivalent range velocity, acceleration, and jerk. The major properties of the proposed method include the following:

- The proposed technique achieves precise RM compensation while avoiding the BSSL effect;
- It reduces the parameter search space from high dimensions to one dimension, and thus has a relatively low computational complexity;
- Through the introduction of a 1-D lag-time variable, the LVD operation improves the signal accumulation gain and enhances the noise robustness;
- The proposed approach demonstrates effective suppression of cross-term interference and enables simultaneous detection of multiple targets.

In general, the proposed method achieves an optimal balance among computational complexity, detection capability, and estimation accuracy. Simulation results involving single-target focusing, multiple moving target detection, and comprehensive performance analyses validate the effectiveness of the presented approach.

This paper investigates long-time coherent integration under a Gaussian white noise background. However, actual detection environments are often more complex due to the ground medium variability, such as post-rainfall moisture fluctuations [35] and complex subsurface formations [36], which introduce significant clutter interference. Therefore, clutter suppression and its impact on long-time coherent integration remain critical issues worthy of further research. 

## Figures and Tables

**Table 1:** Computational complexities of different algorithms.

Methods	Computational Complexity	Search Dimension
RFRT-SLVD	$O[NM+N_{a_{3}}\left(5N_{\tau }M\log_{2}M+N_{\tau }M\log_{2}N_{\tau }\right)+2N_{am}\left(NM\log_{2}M+MN\log_{2}N\right)]$	1-D search
TRT-SKT-LVD	$O[NM+MN^{2}+NM\log_{2}N+N_{am}N\left(5N_{\tau }M\log_{2}M+N_{\tau }M\log_{2}N_{\tau }\right)]$	2-D search
GRFT	$O[N_{a_{1}}N_{a_{2}}N_{a_{3}}NM+N_{a_{1}}N_{a_{2}}N_{a_{3}}N\left(M-1\right)]$	4-D search
ACCF iteratively	$O[3NM\log_{2}N+3NM\log_{2}M+4MN]$	without search

**Table 2:** Radar system parameters.

Parameter	Value
Carrier frequency	5 GHz
Bandwidth	10 MHz
Pulse duration	50 μs
Pulse repetition frequency	1 kHz
Sample frequency	60 MHz
Accumulation time	1 s
Nearest slant range	2 km
Flight velocity of radar	120 m/s

**Table 3:** The motion parameters of a ground maneuvering target.

Parameter	Value
Along-track velocity	10m/s
Cross-track velocity	−45m/s
Along-track acceleration	$2\;m/s^{2}$
Cross-track acceleration	$1\;m/s^{2}$

**Table 4:** The motion parameters of multiple targets.

Parameter	Target 1	Target 2
Nearest slant range (km)	2	1.2
Along-track velocity (m/s)	10	10
Cross-track velocity (m/s)	−45	48
Along-track acceleration ($m/s^{2}$)	5	−7
Cross-track acceleration ($m/s^{2}$)	1	3
SNR after PC (dB)	12	12

**Table 5:** The comparisons between setting parameters and estimated parameters.

Parameters	Velocity (m/s)		Acceleration (m/s^2^)		Jerk (m/s^3^)		Doppler Ambiguity	
	True Value	Estimated Value	True Value	Estimated Value	True Value	Estimated Value	${\hat{M}}_{amb1}$	${\hat{M}}_{amb2}$
Target 1	45	44.9962	3.5333	3.5323	−0.3043	−0.3	1	1
Target 2	−48	−48.0068	3.5417	3.5398	0.5225	0.5	−2	1

## Data Availability

The data presented in this study are available on request from the corresponding author.
